# Improvement of *Vicia faba* plant tolerance under salinity stress by the application of thiamine and pyridoxine vitamins

**DOI:** 10.1038/s41598-024-72511-y

**Published:** 2024-09-27

**Authors:** Eman Zakaria Ahmed, Amira Mohamed Abd El Sattar

**Affiliations:** Botany and Microbiology Department, Faculty of Science, Helwan University, Cairo, Egypt

**Keywords:** *Vicia faba*, Salinity, Thiamine, Pyridoxine, Antioxidants, Vitamins, Physiology, Plant sciences

## Abstract

Enhancement of plant growth at early growth stages is usually associated with the stimulation of various metabolic activities, which is reflected on morphological features and yield quantity and quality. Vitamins is considered as anatural plant metabolites which makes it a safe and ecofriendly treatment when used in appropriate doses, for that this research aimed to study the effect of two different vitamin B forms (thiamine and pyridoxine) on *Vicia faba* plants as agrowth stimutator in addition to study it’s effect on plant as astrong antioxidant under salinity stress.Our findings demonstrated that both vitamin forms significantly increased seedling growth at germination and early growth stages, especially at 50 ppm for pyridoxine and 100 ppm for thiamine. Pyridoxine at 50 ppm increased seedling length by approximately 35% compared to control, while thiamine at 100 ppm significantly promoted seedling fresh and dry wt by 4.36 and 1.36 g, respectively, compared to control seedling fresh wt 2.17 g and dry weight 1.07 g. Irrigation with 100 mM NaCl had a negative impact on plant growth and processes as well as the uptake of several critical ions, such as K^+^ and Mg^+2^, increasing Na uptake in comparison to that in control plants. Compared to control plants irrigated with NaCl solution, the photosynthetic pigments, soluble sugars, soluble proteins, and total antioxidant capacity increased in the presence of pyridoxine and thiamine, both at 50 and 100 ppm salinity. The proline content increased in both treated and untreated plants subjected to salt stress compared to that in control plants. Thiamine, especially at 50 ppm, was more effective than pyridoxine at improving plant health under saline conditions. An increase in *Vicia faba* plant tolerance to salinity was established by enhancing antioxidant capacity via foliar application of vitamin B through direct and indirect scavenging methods, which protect cell macromolecules from damage by oxidative stress, the highest antioxidant capacity value 28.14% was recorded at 50 ppm thiamine under salinity stress.The provided results is aguide for more researches in plant physiology and molecular biology to explain plant response to vitamins application and the suggest the sequence by which vitamins work inside plant cell.

## Introduction

Broad bean or faba bean (*Vicia faba*) is an important edible crop in Egypt. It was first cultivated and utilized as a human food and stock feed in North Africa and Southwest Asia, where it is a member of the Fabaceae family^1^. Currently, faba beans are grown practically everywhere in the world. Most agricultural research focus on enhancing plant growth and productivity or helping plants adapt to various conditions^2^. The nutritional, agronomic, and financial benefits of faba beans are receiving increasing amounts of attention. This legume has a high protein content and a well-balanced amino acid profile,with the exception of low levels of methionine and cysteine^3^. However, lysine is particularly abundant^4^. Additionally, it is a plentiful source of additional healthy nutrients, such as dietary fibers^5^, ash^6^, and phenolic compounds^2,7^. Due to its reduced endogenic lipoxygenase activity^8^ and low lipid content^9^, faba beans are also less likely to acquire off tastes than soybeans and peas.

One of the most common abiotic stresses affecting plant physiology is salinity, caused by increase of NaCl concentration in soil^10–12^. Several plant problems (nutrient ion imbalance, reduction in stomatal conductance, and reduced photosynthetic activity) are caused by salt stress^13,14^. Osmotic stress, causes toxicity by increasing Na + and Cl^-^ ions concentrations as well as increasing ROS production that cause oxidative stress^15^, secondary metabolite alterations (signal molecules, hormones, and oxidant chemicals), as well as morphological alterations (decreases in leaf number, plant size, root length, and fruit output)^12,16^. According to several studies on various plants, NaCl stress decreases plantsfresh and dry weights of roots and shoots^17–20^.

Vitamins play a crucial role in both plant and animal metabolism; hence, supplementation with vitamin B in various forms may be a suitable first step in increasing plant tolerance to abiotic conditions such as salt. Because of their redox chemistry, role as cofactors, and strong antioxidant potential^21–26^. Studies on the application of vitamin B forms on plants is not enough and more researches are needed in this point to explain it’s vital role in plant and make use of it in agriculture field.

Thiamine, also known as vitamin B1, was the first vitamin B identified^27^. Free thiamine, thiamine monophosphate (TMP) and thiamine pyrophosphate (TPP) are the three most predominant forms of B1 that exist in cells^28^.

Thiamine is a colorless, water-soluble vitamin that is predominantly produced by plants and microorganisms and is crucial for human nutrition^29^.Thiamine is widely distributed across plant organs, namely, leaves, flowers, fruits, seeds, roots, tubers and bulbs^21^. Thiamine pyrophosphate, the cofactor form of vitamins, is by far the most prevalent form of vitamin B1 in plants. It is a crucial component required in many metabolic activities, such as acetyl-CoA biosynthesis, amino acid biosynthesis, the Krebs cycle, and the Calvin cycle^30^. Thiamine has been shown to alleviate the effects of several environmental stresses on *Arabidopsis* (*Arabidopsis thaliana*), presumably by protecting the plant from oxidative damage^31^. Because oxidative stress is required for the renewal of antioxidants such as glutathione and ascorbate, thiamine may indirectly function as an antioxidant in plants by supplying NADH and NADPH to counteract these conditions^21,32,33^ also play a significant role in the transketolation processes of the pentose phosphate cycle, which provides pentose phosphate for nucleotide synthesis and produces the reduced form of NADP required for several metabolic pathways^34,35^. However, thiamine also functions as a coenzyme in the decarboxylation of keto acids such as pyruvic acid and keto-glutamic acid and plays a role in carbohydrate and fat metabolism^30^. Thiamine is a powerful scavenger of superoxide anions and hydroxyl radicals^36^ that helps in protecting membranes from lipid peroxidation. Numerous studies have revealed that the application of thiamine to plants increases their vegetative growth and chemical content. The growth of mustard plants was significantly accelerated by soaking seeds in thiamine hydrochloride.

Thiamine builds up in plants under various abiotic conditions, and its exogenous administration confers a degree of tolerance to salt and oxidative stresses^23,37^. Indeed, Sayed and Gadallah^38^ demonstrated that the beneficial effects of thiamine administered topically to shoots or subcutaneously to roots of sunflower plants were offset by salt stress. In comparison to those of untreated plants, thiamine-treated plants under salt stress presented higher chlorophyll levels, greater relative water contents, lower leaf water potential, higher concentrations of soluble sugars and total free amino acids, lower concentrations of Na^+^, Ca^+2^, and Cl^-^, and higher concentrations of K^+^. This finding is in agreement with the findings of Fallahi et al.^39^, who showed that foliar thiamine spraying, particularly at a concentration of 750 M, improved vegetative growth, leaf nutrient content (N, P, and Ca^+2^), and chlorophyll content in basil plants. The same results were also observed in *Vicia faba*^40^, *Oryza sativa*^41^, and *Lupinus termis*^42^. Similarly, research on maize showed that foliar application of thiamine (100 ppm) increased the number of green leaves, the leaf area index (LAI), and postponed leaf senescence. In comparison to the corresponding control, thiamine also increased the antioxidant capacity^43^.

Vitamin B6 comprises a group of compounds that include pyridoxine, pyridoxal, pyridoxamine, and their phosphorylated derivatives. All organisms require the important metabolite pyridoxine (vitamin B6) in its active form, pyridoxal 5′-phosphate^44^. Numerous metabolic enzymes may utilize it as a coenzyme, and recent research has revealed that it is also an effective antioxidant^45^. A de novo synthesis pathway for vitamin B6 exists in both plants and microbes. It has been found that some plant species need it for growth and differentiation^46^.

Foliar treatment may be a useful option for these plants since, while certain plant species’ roots can synthesize vitamin B, the roots of other plant species cannot^47^ and are dependent on transfer from the shoot^48^. Many metabolic enzymes, such as those involved in the metabolism of amino acids and the production of antibiotics, are crucial cofactors. The pyridoxine (vitamin B6) concentration increased to some extent (1750 mg) according to Mahdi et al.^49^.Desouky^50^ and Hamada and Khulaef^40^ showed that soaking *Vicia faba* seeds in pyridoxine (vitamin B6) stimulated both photosynthetic pigment and the net photosynthetic rate. Similarly, Khan et al.^51^ noted that applying vitamin B6 to wheat plants at a particular limit resulted in the highest increase in growth parameters.

The objective of this study was to assess the physiological impact of two forms of vitamins (B) thiamine and pyridoxine, on *Vicia faba* vegetative growth under salt stress in order to improve plant tolerance to stress.

## Materials and methods

**Fig. 1:**
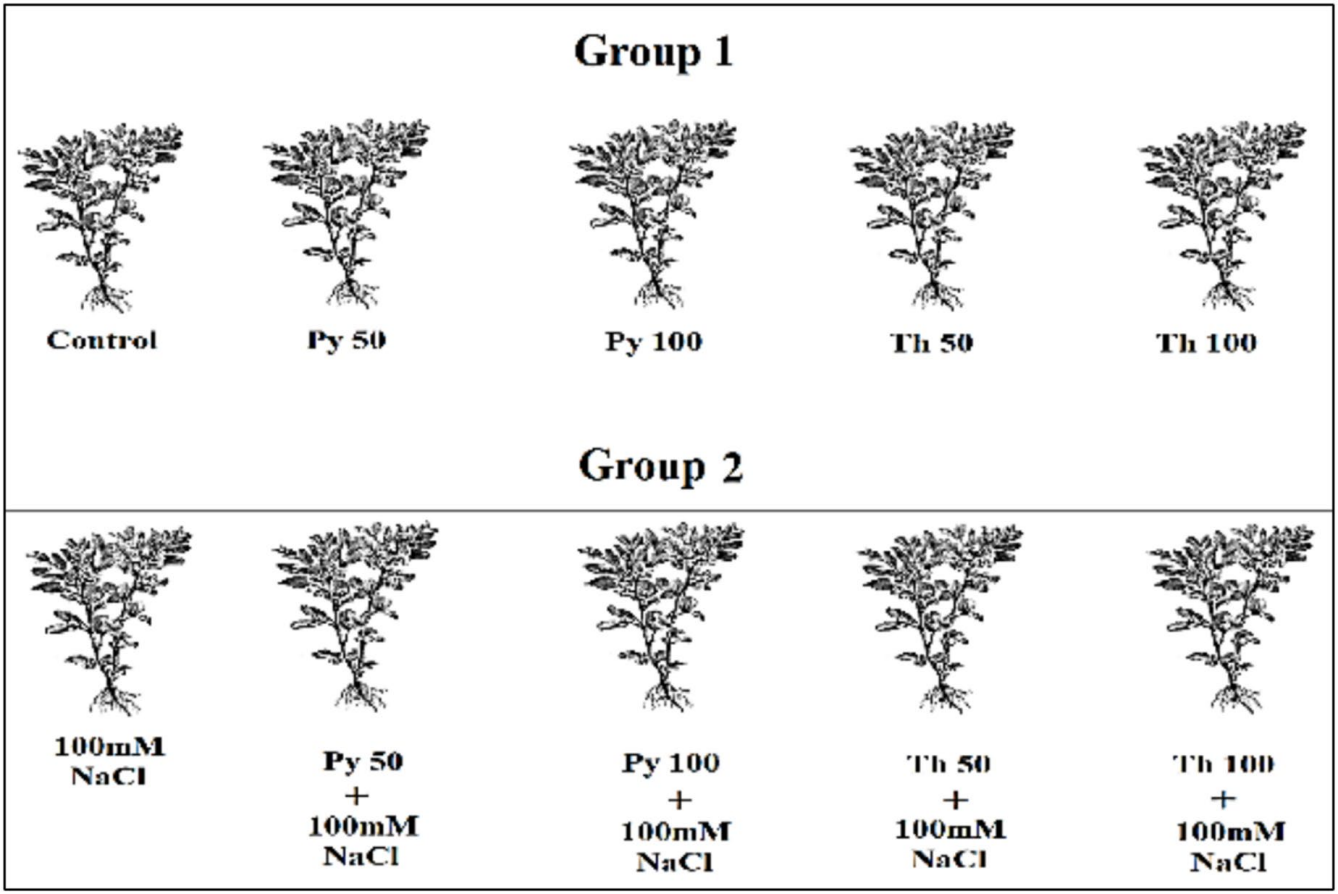
Graphical figure representing the design of pot experiment and showing different groups treatments of application of Py (Pyridoxine) Th (Thiamine) both at 50 and 100 ppm on *Vicia faba* plants grown under normal conditions and salinity stress at100mM NaCl.

In March 2022, a pot experiment established at the Helwan University farm. Broad bean seeds were supplied by Egypt’s Giza Agricultural Research Centre. First, a preliminary experiment was performed on bean seeds to study the response to a wide range of pyridoxine and thiamine concentrations. Seeds were soaked in pyridoxine and thiamine (0, 50, 100, 150, 200, 250, 300 ppm) for 12 h and subsequently sown in small pots filled with clay, with 6 replicates for each treatment and five seeds per pot. After 2 weeks,the seedling length and fresh and dry weights were measured. From these preliminary experimental results, the most effective concentrations were selected for the pilot experiment. In pots filled with loamy soil, uniform bean seeds were planted, and 21 days after planting, the plants were divided into two groups: one group was irrigated with tap water, and the other group was irrigated with 100 mM NaCl solution. The plants in each group were divided into five subgroups (Fig. 1), each represented by five pots containing three seedlings. Pyridoxine and thiamine were applied as foliar sprays twice on the leaves at 50 and 100 ppm, and distilled water was applied as a foliar spray for controls.

### Growth criteria

Two weeks after the last foliar spray, the stem and root length, shoot and root fresh and dry weights and number of leaves were recorded, and samples were taken for chemical analysis.

### Photosynthetic pigment content

The method of Metzener et al.^52^ was used to assess the photosynthetic pigment content in faba bean leaves. The concentrations of chlorophyll a and b, total chlorophyll, and carotenoids were calculated using the following equations:

Chl. *a* = 10.3 E_664_- 0.918 E_645_.

Chl. *b* = 19.7E_645_-3.87E_664_.

Total chlorophyll (a + b) = Chl a + Chl b.

Carotenoids = 4.3 E_452_ (0.0265 Chl.*a* + 0.426 Chl.*b*).

### Total soluble sugars

As described by Umbriet et al.^53^,theanthrone approach was used to measure the total amount of soluble sugars. Three milliliters of sample was treated with six milliliters of anthrone solution (2 g/L H_2_SO_4_ 95%) and kept in a boiling water bath for three minutes.After cooling, the generated color was spectrophotometrically measured at 620 nm.

### Total soluble proteins

Total soluble protein was measured according to the methods of Lowry et al.^54^. Briefly, 2% sodium carbonate in 4% sodium hydroxide, 0.5% copper sulfate in 1% sodium tartrate, and 1 ml of faba bean extract were combined in a freshly mixed solution (50:1 v/v). After standing for 10 min, the mixture was diluted to a specific volume with 0.5 ml of Folin-Phenol reagent (1:3). After 30 min, the optical density of the mixture was measured at 750 nm.

### Proline

Ten milliliters of sulfo-salicylic acid (3%) was used to homogenize approximately 0.5 g of faba bean leaves before filtering the mixture using Whatman No. 1 filter paper. Using the technique outlined by Bates et al.^55^, free proline was measured. From a standard curve, the proline concentration was calculated as mg proline/g dry weight.

### Phosphomolybdenum assay

The antioxidant activity of the fractions was assessed using the phosphomolybdenum method following the methods of Prieto et al.^56^. One milliliter of the reagent solution (0.6 M sulfuric acid, 28 mM sodium phosphate, and 4 mM ammonium molybdate) was added to an aliquot of 0.1 ml of each fraction that had been dissolved in its corresponding solvent. The vial was sealed and left to sit at 95 ℃ in a water bath for 90 min. The samples were cooled to room temperature after incubation, and the absorbance of the mixture at 765 nm was measured in comparison to that of the control. The following formula was used to determine the percent inhibition, and the software Graph Prism Pad was used to determine the IC50.

% inhibition = (1– absorbance of sample/absorbance of control) × 100.

### Mineral ions

A 15 ml acid mixture of HNO_3_: HCl (1:1, v/v) was used to digest approximately 0.5 g of oven-dried leaves, and the digest was heated on a hot plate until it was clear. The digest was double deionized water diluted to 25 ml, cooled, and filtered. At the Ecology Lab, Helwan University’s Faculty of Science, phosphorus, potassium, sodium and magnesium concentrations were measured using microwave plasma atomic emission spectroscopy (Agilent Technologies 4210 MP-AES). Mineral ions were expressed as ppm on a dry matter basis.

### Statistical analysis

The least significant difference (LSD at the 5% level) was used to determine statistical significance for the compared means via one-way analysis of variance (ANOVA) and Duncan’s multiple comparison test using IBM Statistical Version 21.

## Results

**Fig. 2:**
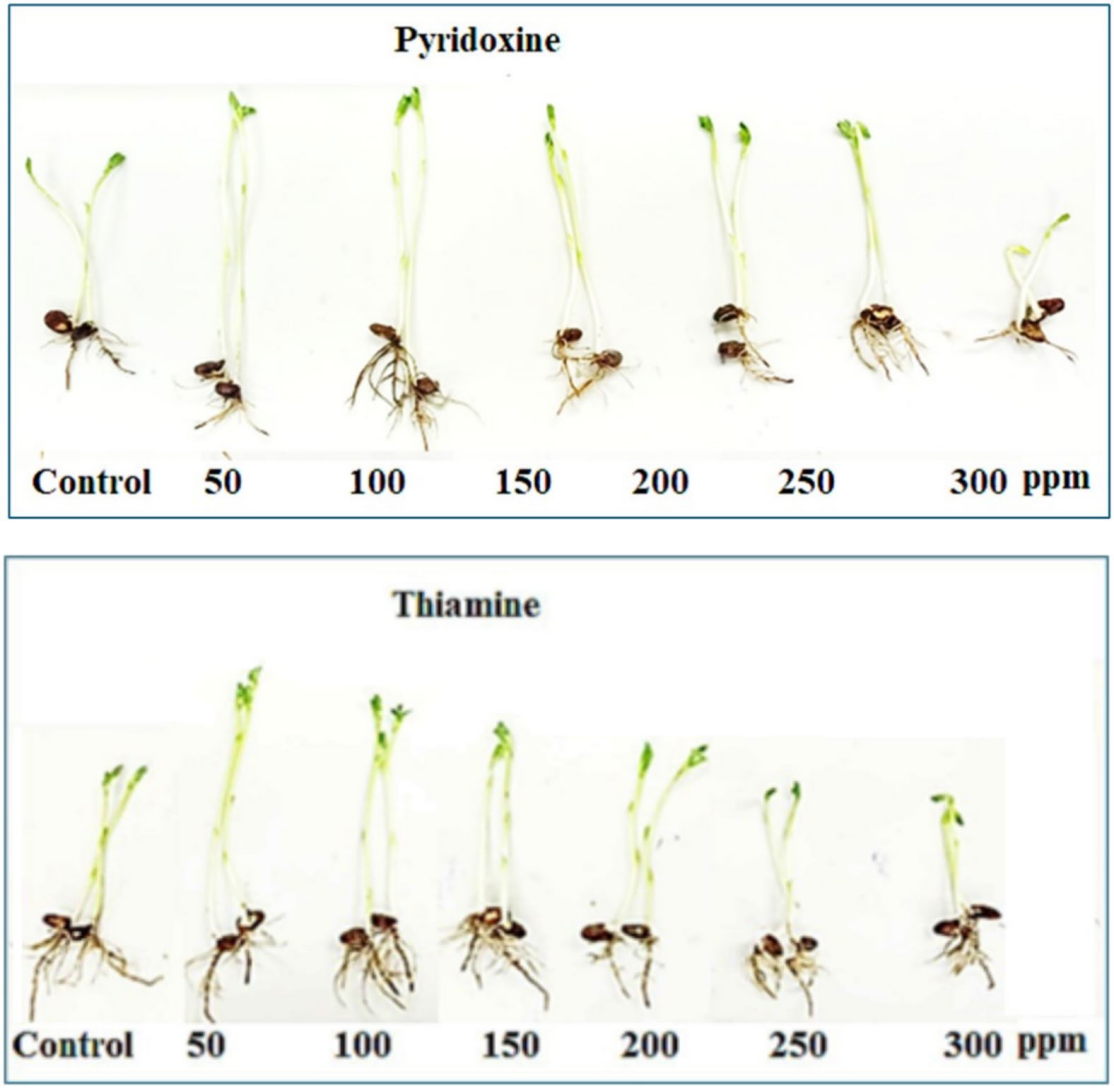
Effect of (**a**) Pyridoxine **(b**) Thiamine at awide range of concentrations between (50 ppm –300 ppm) on *Vicia faba* plant seedling growth.

The preliminary experiment results (Table 1 and Fig. 2) showed that soaking faba beans seeds in pyridoxine at concentrations up to 250 ppm and thiamine at concentrations up to 200 ppm significantly improved the growth criteria of bean plants, represented by shoot and root length and fresh and dry weight. Compared with corresponding control plants, the most effective concentrations of both vitamin B forms were 50 and 100 ppm. Pyridoxine at 50 ppm was more effective than thiamine at increasing seedling length by approximately 35%, while thiamine at 100 ppm significantly promoted seedling fresh and dry wt by 4.36 and 1.36 g, respectively, compared to that of the control seedling fresh wt 2.17 g and dry weight 1.07 g.

**Table 1:** Effect of two different vitamin B forms on vegetative growth characteristics of *Vicia faba* seedling growth.

Treatments	Shoot length (cm)	Root length (cm)	Seedling fresh wt. (g)	Seedling dry wt. (g)
Control	14.5 ± 0.27 e	7 ± 0.12 b	2.17 ± 0.039 g	1.07 ± 0.012 d
Py 50	21 ± 0.36 a	8 ± 0.22 a	3.72 ± 0.061 bc	1.32 ± 0.025 b
100	20 ± 0.38 b	8 ± 0.14 a	3.63 ± 0.059 ef	1.22 ± 0.020 cd
150	18 ± 0.33 c	6 ± 0.11 c	3.46 ± 0.058 cd	1.16 ± 0.017 d
200	16 ± 0.30 d	5 ± 0.092 d	3.07 ± 0.055 de	1.08 ± 0.015 e
250	15.5 ± 0.29 de	5 ± 0.14 d	2.58 ± 0.045 f.	1.03 ± 0.011 e
300	7.5 ± 0.13 h	4.5 ± 0.082 d	2.02 ± 0.039 g	0.79 ± 0.01 f.
Th 50	20 ± 0.33 b	7 ± 0.18 b	4.14 ± 0.076 ab	1.23 ± 0.021 c
100	18 ± 0.37 c	7 ± 0.24 b	4.36 ± 0.079 a	1.36 ± 0.020 a
150	15.5 ± 0.29 de	7 ± 0.19 b	3.85 ± 0.070 bc	1.17 ± 0.018 cd
200	15.5 ± 0.24 de	7 ± 0.19 b	3.74 ± 0.067 bc	1.17 ± 0.018 cd
250	13.5 ± 0.24 f.	6 ± 0.11 c	3.03 ± 0.057 de	1.18 ± 0.019 cd
300	10 ± 0.18 g	6 ± 0.074 c	2.17 ± 0.034 h	1.17 ± 0.019 cd
L.S.D at 5%	0.96	0.5	0.41	0.09

Data shown in the table represent the mean ± standard error, followed by a small letter; similar letters indicate that means were not different significantly at 5%, probability based on Duncan’s test.

The main pot experiment data presented in Table 2 show that, compared to control plants, the tested pyridoxine and thiamine concentrations increased all the measured growth parameters in *Vicia faba,*represented by stem and root length;the fresh and dry weights of shoots and roots; and the number of leaves. The highest growth parameters were recorded for 100 ppm thiamine, followed by 50 ppm pyridoxine. Salinity negatively affected the morphological growth of *Vicia faba*plants compared to the corresponding control plants. In addition to the promotive effect of vitamins under normal conditions, there was a significant increase in all growth criteria under salt stress compared to those in untreated salt-stressed plants.

**Table 2:** Effect of two different vitamin B forms on vegetative growth characteristics of *Vicia faba* plant grown under salinity stress.

Treatments	Shoot length (cm)	Root length (cm)	Shoot fresh wt (g)	Soot dry wt. (g)	Root fresh wt. (g)	Root dry wt. (g)	No of leaves
0.0	25.5 ± 0.49 cd	22 ± 0.42 bc	9.56 ± 0. .17 d	0.96 ± 0.015 d	4.63 ± 0.07 de	0.33 ± 0.013 de	6 ± 0.092 b
NaCl (100 mM)	21.3 ± 0.39 e	15.5 ± 0.27 e	6.17 ± 0.11 g	0.66 ± 0.012 f.	2.21 ± 0.06 f.	0.21 ± 0.012 e	5 ± 0.09 c
Py 50	27.5 ± 0.60 b	25.3 ± 0.49 a	13.70 ± 0.25 a	1.17 ± 0.020 ab	7.82 ± 0.14 a	0.59 ± 0.016 b	7 ± 0.12 b
Py 50 + NaCl (100 mM)	25 ± 0.48 cd	20.4 ± 0.37 d	7.71 ± 0.13 ef	0.93 ± 0.015 d	4.12 ± 0.07 e	0.56 ± 0.015 bc	6 ± 0.091 a
Py 100	26.3 ± 0.52 cd	25.5 ± 0.49 a	11.72 ± 0.21 c	1.10 ± 0.020 c	7.47 ± 0.14 c	0.59 ± 0.016 bc	8 ± 0.014 a
Py 100 + NaCl (100 mM)	24.5 ± .43 cd	21.4 ± 0.38 bcd	8 ± 0.14 b	0.71 ± 0.012 ef	5.50 ± 0.10 ef	0.44 ± 0.014 cd	7 ± 0.12 a
Th 50	26 ± 0.50 bc	22.5 ± 0.40 a	11.71 ± 0.20 c	1.12 ± 0.017 bc	6.10 ± 0.12 bc	0.64 ± 0.017 b	8 ± 0.14 a
Th 50 + NaCl (100 mM)	23.8 ± 0.42 d	21.0 ± 0.38	7.41 ± 0.13 f.	0.72 ± 0.012 e	5.00 ± 0.09 cde	0.51 ± 0.015 bc	7 ± 0.12 a
Th 100	30 ± .85 a	25 ± 0.49 a	12.61 ± 0.23 b	1.18 ± 0.03 a	7.87 ± 0.14 a	1.05 ± 0.12 a	8 ± 0.14 a
Th 100 + NaCl (100 mM)	25.7 ± 0.55 ab	22.2 ± 0.42 a	7.86 ± 0.17 ef	0.81 ± 0.013 f.	5.89 ± 0.08 bc	0.58 ± 0.016 bc	7 ± 0.12 a
L.S.D at 5%	2.1	1.3	0.9	0.18	1.3	0.06	1

Data shown in the table represent the mean ± standard error, followed by a small letter; similar letters indicate that means were not different significantly at 5%, probability based on Duncan’s test.

Foliar spraying with vitamin B alleviated the negative effect of salinity on essential ion uptake and controlled the uptake of Na^+^ ions. Thiamine and pyridoxine foliar sprays on *Vicia faba* enhanced the absorption of the mineral ions Mg andK, while the sodium uptake decreased in these plants compared to that in untrated plants affected by salinity.The best concentration was 100 ppm for both vitamin B forms (Table 3).

**Table 3:** Effect of two different vitamin B forms on mineral ions contents (mg g^−1^d.m) of *Vicia faba* plant grown under salinity stress.

Treatments	Mg	K	Na	K/Na ratio
0.0	0.714 ± 0.013 bcd	3.38 ± 0.06 d	2.10 ± 0.039 c	1.60 ± 0.020 d
NaCl (100 mM)	0.669 ± 0.012 d	3.00 ± 0.05 e	3.12 ± 0.057 a	0.96 ± 0.017 e
Py 50	0.792 ± 0.014 a	4.21 ± 0.09 b	1.91 ± 0.035 d	2.30 ± 0.041 c
Py 50 + NaCl (100 mM)	0.704 ± 0.013 bc	3.45 ± 0.07 d	2.33 ± 0.042 b	1.48 ± 0.019 d
Py 100	0.718 ± 0.013 bcd	4.54 ± 0.094 a	1.10 ± 0.020 g	4.12 ± 0.080 a
Py 100 + NaCl (100 mM)	0.701 ± 0.012 cd	3.58 ± 0.075 cd	2.12 ± 0.037 c	1.68 ± 0.025 d
Th 50	0.730 ± 0.015 bc	4.05 ± 0.08 b	1.48 ± 0.027 e	2.73 ± 0.062 b
Th 50 + NaCl (100 mM)	0.699 ± 0.013 cd	3.45 ± 0.07 d	2.20 ± 0.037 c	1.56 ± 0.023 d
Th 100	0.756 ± 0.014 ab	3.74 ± 0.065 c	1.31 ± 0.024 f.	2.85 ± 0.050 b
Th 100 + NaCl (100 mM)	0.700 ± 0.013 cd	3.55 ± 0.055 cd	2.23 ± 0.039 b	1.52 ± 0.020 d
L.S.D at 5%	0.04	0.31	0.13	0.23

Data shown in the table represent the mean ± standard error, followed by a small letter; similar letters indicate that means were not different significantly at 5%, probability based on Duncan’s test.

**Fig. 3:**
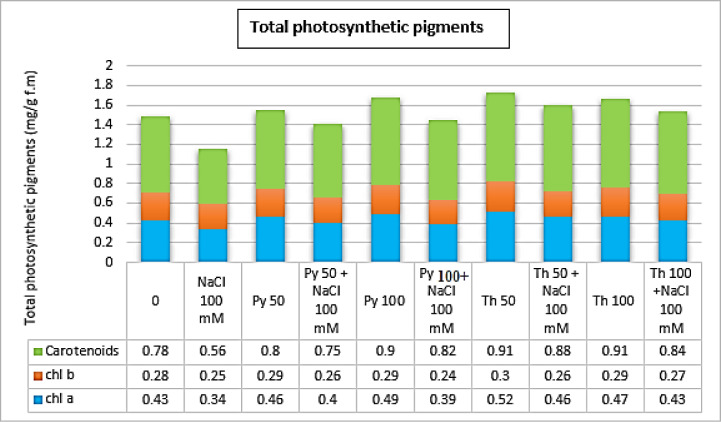
Effect of two different vitamin B forms: Py (Pyridoxine) Th **(**Thiamine) on Total photosynthetic pigment of *Vicia faba* plant grown under salinity stress. Values represent the mean of three replicates. Different letters (a, b, c, d, e and f) indicate statistical differences at 5% probability according to Duncan’s test. Error bars are standard errors of the mean.

The data presented in Fig. 3 show that salinity decreased the total photosynthetic pigment content (1.15 mg g^−1^d.m.) compared to the corresponding control (1.49 mg g^−1^d.m.). The application of pyridoxine and thiamine at 50 and 100 ppm increased the chlorophyll a, b and carotenoid contents in faba bean leaves under normal and salt-stressed conditions, especially at 50 ppm thiamine in both normal plants and plants subjected to salinity stress (1.73 and 1.6 mg g^−1^d.m.), respectively.

**Fig. 4:**
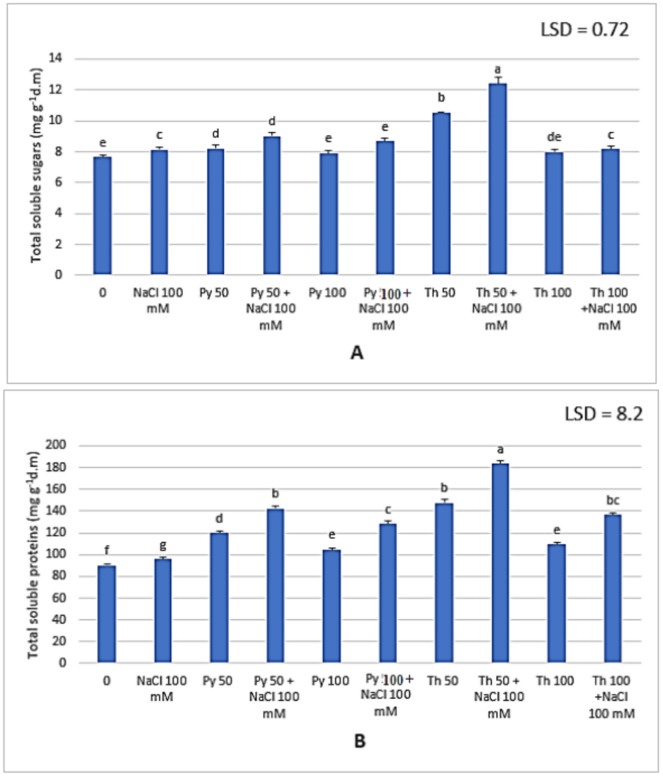
Effect of two different vitamin B forms : Py (Pyridoxine) Th (Thiamine) on (**A**) total soluble sugars and (**B**) total soluble protein contents of *Vicia faba* plant grown under salinity stress. Values represent the mean of three replicates. Different letters (a, b, c, d, e, f an g) indicate statistical differences at 5% probability according to Duncan’s test. Error bars are standard errors of the mean.

**Fig. 5:**
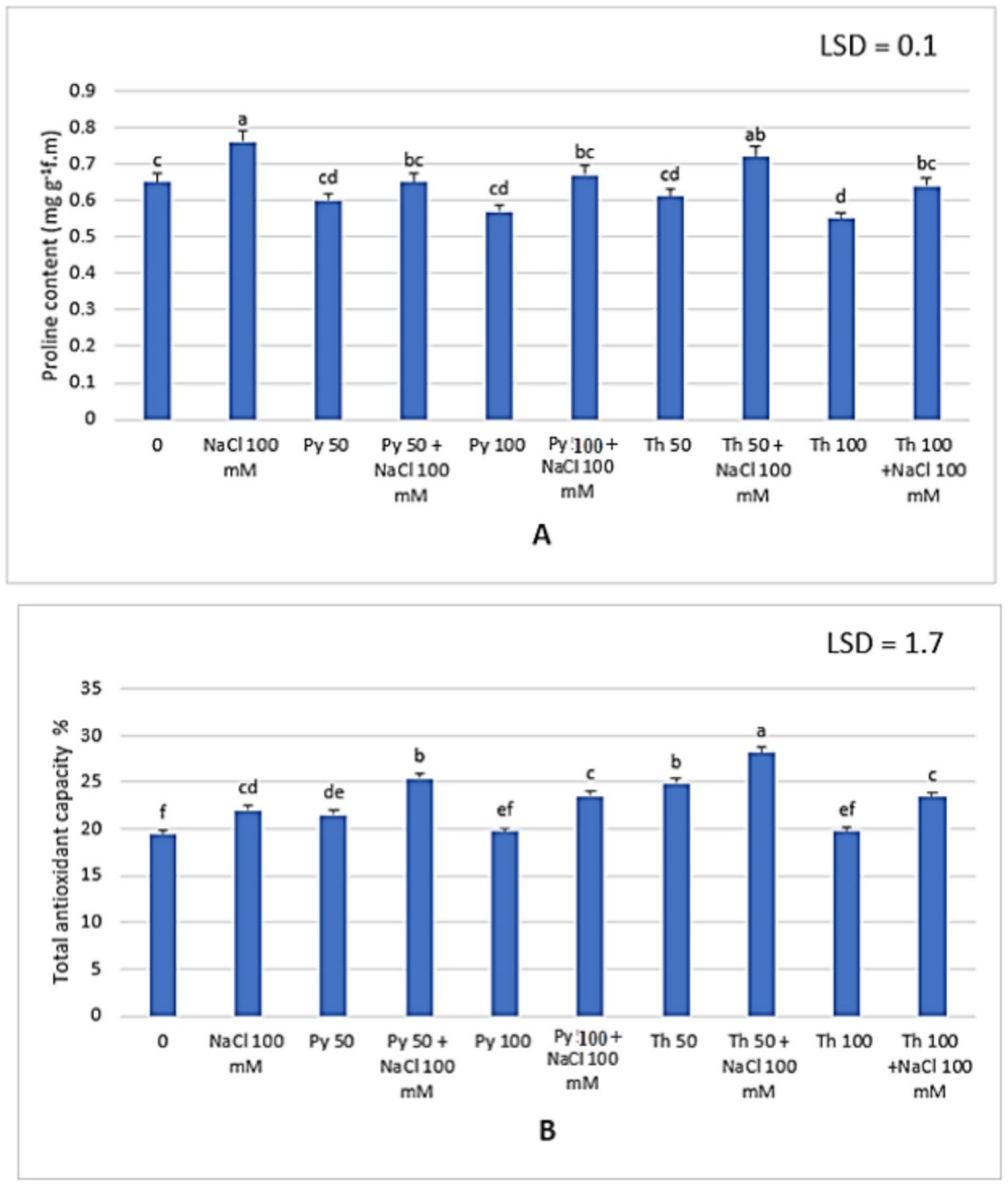
Effect of two different vitamin B forms on : Py (Pyridoxine) Th (Thiamine) (**A**) Proline content and (**B**) Total antioxidant capacity % of *Vicia faba* plant grown under salinity stress. Values represent the mean of three replicates. Different letters (a, b, c, d, e and f) indicate statistical differences at 5% probability according to Duncan’s test. Error bars are standard errors of the mean.

The effect of salinity on photosynthetic pigments is strongly related to plant primary metabolites and the ratio of soluble to insoluble contents in plants. Soluble sugars and proteins accumulated under saline conditions in faba bean leaves compared to those in control plants. In addition, vitamin B in both forms significantly increased the soluble sugar and protein contents compared to those of the control and untreated salt-stressed plants. The highest content was recorded at 50 ppm thiamine in normal and salt-stressed plants (10.56 and 12.4 mg g^−1^d.m soluble sugars) and (147.57 and 183.33 mg g^−1^d.m for soluble proteins) (Fig. 4). Proline, also known as a stress marker, increased under 100 mM NaCl. Vitamin B in both forms helped plants overcome stress, and there was a significant decrease in proline content (Fig. 5A). The lowest proline content was 0.55 g^−1^d.m at 100 ppm thiamine under normal conditions compared to that in control plants 0.65 g^−1^d.m. Treatment decreased the proline content in the salt-stressed plants from 0.76 g^−1^d.m to approximately the same level as that in the control leaves.

Total antioxidant capacity is an important screen for evaluating plant tolerance. Under salinity, the total antioxidant capacity increased to 22%, whereas it was 19.4% for control plants. After foliar application of both thiamine and pyridoxine, the antioxidant capacity increased both under normal conditions and under 100 mM NaCl, with the highest value reaching 28.14% recorded at 50 ppm thiamine (Fig. 5B). Both vitamin B forms helped *Vicia faba* plants tolerate salinity, while thiamine was more effective than pyridoxine.

## Discussion

Vitamins use in maintenance of plant growth, development and adaptation may be asuitable solution in agriculture for many severe problems affecting plants productivity nowadays. Pyridoxine and thiamine significantly promoted *vicia faba* growth at both early seedling stage and vegetative growth under both normal and salt stress conditions. The findings of the present study on *vicia faba* plant are in agreement with those of prior research showing Na toxicity in wheat^55^, *Pisum sativum* L.^56^, *vicia faba*^57^, chickpea species^58^,and *Oenanthe javanica* species^59^. Increased Na^+^ and Cl^-^ concentrations in plants are a result of soil salinity, which also affects the ratio of Na^+^/K^+^ and the regular ionic activities of plants^60^. This increase in osmotic stress decrease water uptake and transport. The hormone-induced sequential responses that result from water uptake inhibition might decrease stomatal opening, carbon dioxide assimilation, and the photosynthetic rate^60,61^. A decrease in nitrate reductase activity, inhibition of photosystem II^62^, and chlorophyll breakdown^63,64^ have been also observed. A reduction in the efficiency of photosynthesis and carbon gains and a shift in energy from growth to the homeostasis of salt stress could also contribute to a decrease in growth^59,63^. Photosynthesis is impacted by high NaCl concentrations, and longer-term salt stress reduces the production of the chlorophyll protein-lipid complex^65^.

Salinity reduces the production of new proteins that connect chlorophyll by enhancing the activity of the enzyme chlorophyllase^66^. During this process, K + is a known enzyme stimulant for a number of photosynthesis-related enzymes. Therefore, decreases in K + levels prevent photosynthesis, which ultimately results in decreased growth^67^. In addition to increasing antioxidant capacity through enzymatic and nonenzymatic mechanisms, plants overcome oxidative damage by accumulating osmoregulatory and osmoprotectants such as soluble sugars and soluble proteins such as proline, which are key stress markers. This enables plants to tolerate stress and protects them from harm caused by unchecked ROS production.

Vitamins are regarded as vital antioxidants in plants, and exogenous vitamin administration may work in conjunction with endogenous vitamin levels to help plants withstand and respond to environmental challenges. Vitamins had positive effects on plant nutrition, and growth certainly influenced the yield of the components. Mainly vitamin B positive effect is generally related to it’s high antioxidant and protective role on plant macromolecules, improvemevt of chlorophyll content, hormonal and nutrient balance, all these effects improves plant metabolism during seed germination and vegetative growth.

B-group vitamins are quite effective at increasing ion absorption^68^. Similar findings were made by Youssef and Talaat^69^, who reported that foliar thiamine enhanced the contentof total nitrogen, phosphorus, and potassium in rosemary plants. Thiamine applied topically to leaves has been shown to help mustard plants better absorb nitrogen^70^. Abd El-Aziz et al.^71^ also stated an increase in phosphorus and nitrogen uptake. The function of thiamine in dividing meristematic stem cells and organ starting cells may be related to its role in promoting basal growth and seed development, according to Martinis et al.^72^.In addition, it plays a role in the growth and division of cells, the production of nucleic acids, and the control of the assimilation of carbon^73^. Thiamine may protect cell membranes and their binding transporters, which leads to increased absorption and translocation of minerals^38,74^. In addition, an increase in nutrient solubility in the rhizosphere of vitamin-treated plants through the secretion of organic acids into the soil is another reason for increased nutrient uptake by the plant^71^.

A notable increase in plant height, biomass and chlorophyll content was observed in wheat by thiamine application both under control and water stress conditions. Moreover, reduction in oxidative stress markers viz., hydrogen peroxide (H2O2), malondialdehyde and electrolyte leakage was found with thiamine application under stress. In addition, cellular antioxidants, mineral contents and yield attributes were also enhanced by thiamine application especially at 100 mg/L under water stress^75^ the same result was repoted on *Pisum sativum* L. at 250, 500 ppm^76^.

According to Kaya et al.^77^, increased growth in plants treated with thiamine is linked to decreased membrane ermeability; decreased malondialdehyde (MDA) and H_2_O_2_ levels; altered antioxidant enzyme activities, such as catalase, superoxide dismutase, and peroxidase; and increased photosynthetic pigment and PSII activity. Exogenous thiamine administration increased antioxidant enzyme activity in *Gerbera jamesonii*, according to Mansouri et al.^78^. The results reported by Abdel-Monaim et al.^79^ on soybeans demonstrated that plants treated with thiamine and riboflavin had higher antioxidant enzyme activity than controls, including peroxidase, polyphenol oxidase, and phenylalanine ammonia lyase. These findings could be explained by the function of thiamine in a number of metabolic pathways, including photosynthesis, cellular respiration, and sugar and protein metabolism.Thi application counteracts the inhibitory effect of ROS on chlorophyll content^77^. The promotion of carotenoid synthesis as a protector of chlorophyll against oxidation by Thi is another reason for the increase in chlorophyll concentration in plants treated with Thi^41^. Moreover, the overexpression of the 2-deoxy-D-xylulose-5-phosphate enzyme, which is strongly dependent on Thi-diphosphate, correlates with the accumulation of chlorophyll^31^.

Thiamine may lead to an increase in the accumulation of certain osmoregulatory agents in plant tissues, which may have an impact on water potential and, in turn, may increase the turgor pressure required for cell expansion and, ultimately, plant development^38^. Additionally, proper regulation of photosynthesis and energy generation is purportedly responsible for the improved growth and yield of plants treated with thiamine^72^. Under different abiotic stress the effect of thiamine also was reported, thiamine acid application improves length, biomass, leaf area, photosynthetic pigment and mineral nutrition, and reduces H2O2, Na, and Pb accumulation in roots and shoots under lead stress^80^.

Researches on the effect of pyridoxine on plant are limited compared to thiamine. Dalatabadian and Modarressanavy^81^ reported that growing sunflower plants with pyridoxine concentrations up to 400 ppm resulted in a significant increase in plant dry weight. It appears that pyridoxine also plays a significant role in cell division. Asli and Houshmandfar^82^ reported that soaking seeds in pyridoxine solution might enhance the growth of maize plants. Mahdi et al.^49^ also reported that the application of pyridoxine (vitaminB6) at a certain concentration (1750 mg. L-1can protect wheat plants and increase their resistance to salinity stresses.Treatment of wheat plants with pyridoxine improved cell division, root growth, and nutrient uptake, which improved the efficiency of the photosynthetic surface and increased the formation of dry matter. Additionally, according to Hamada and Khulaef^40^, pyridoxine seed pretreatment and foliar treatments of beans increased the biosynthesis of photosynthetic pigment fractions. Additionally, Hendawy and Ezz El- Din^83^ and Nassar et al.^84^ both supported the elevated levels of photosynthetic pigments found in *Foeniculum vulgare* var. azoricum and sesame plants. The endogenous levels of plant growth regulators have also been demonstrated to be enhanced by vitamins. Endogenous IAA and phenolic levels were markedly elevated by exogenous therapy, such as foliar spraying of thiamine vitamin on lupine plants. These increases could be attributed to pyridoxine’s role in IAA production and its ability to slow the breakdown of the compound^85^. In general, the improvements in ion uptake, growth promoter levels, protection of photosynthetic pigments, and enhancement of plant antioxidant capacity may be the causes of the overall increase in plant vegetative growth caused by the application of vitamin B. All these processes control and regulate plant photosynthetic capacity, which is the primary source of all macromolecules used in plant adaptation to stress.

Osmoregulation is one of the most significant strategies for adapting to salt stress. According to our findings, during salt stress, soluble sugar and protein concentrations in faba bean plants increase with both vitamin types. Reduced photosynthetic pigments caused by salinity have an impact on the generation of sugars during photosynthesis. Instead of growing more, certain tolerant plants store soluble carbohydrates and proteins as an osmoprotectant mechanism to boost water intake and prevent harm from high salt concentrations in soil. In addition to serving as ROS scavengers, these solutes may be crucial storage carbon and nitrogen supplies^86^.

By promoting osmosis in the cytoplasm, balancing proteins and membranes, and maintaining higher water levels needed for plant growth and cell activities, osmoprotectants (total soluble sugars, proline, and free amino acids) significantly impact how well cells adapt to varying unfavorable environmental stresses. An increased TSS enhances cell membrane maintenance and turgor maintenance. The accumulation of proline and soluble sugars under stress conditions protects cells by maintaining the osmotic strength of the cytosol in combination with that of vacuoles and the external environment. In addition to its osmoprotective role, proline is commonly used as a ROS scavenger and provides protection to enzymes and stabilizes their structures^87,88^. Proline accumulation is suggested to be a symptom of stress in various plants, acting as an osmotic barrier and assisting in cell turgor stability^89^. Free amino acid buildup associated with stress can be a part of the adaptive technique that assists with osmotic equilibrium.

## Conclusion

*Vicia faba* is an economically important plant that is sensitive to salinity stress, which is one of the main abiotic factors affecting plant productivity. Increased plant resistance to salt stress may increase the use of agricultural land, which is frequently exposed to high salt concentrations while overcoming yield loss. In addition to improving plant tolerance and yield quality and quantity. Vitamins such as vitamin B(thiamine and pyridoxine) are safe, affordable and inexpensive to farmers. Both vitamin forms improved bean plant growth especially at 50 and 100 pm leading to significant increases in seed germination, vegetative plant growth; photosynthetic pigment content; sugar and protein content;and ion uptake and antioxidant capability in *Vicia faba* plants under normal or salt stress conditions. More physiological and genetical studies are still needed in this point to understand the effect of vitamins based on gene expression and sequence of plant adaptation steps to stress.

## Data Availability

All the data generated or analyzed during this study are included in this article.
